# Effectiveness, Safety, and Tolerability of Nabiximols Oromucosal Spray vs Typical Oral Long-Acting Opioid Analgesics in Patients with Severe Neuropathic Back Pain: Analysis of 6-Month Real-World Data from the German Pain e-Registry

**DOI:** 10.1093/pm/pnab263

**Published:** 2021-09-04

**Authors:** Michael A Ueberall, Carlos Vila Silván, Ute Essner, Gerhard H H Mueller-Schwefe

**Affiliations:** 1 Institute of Neurological Sciences, Nuernberg, Germany; 2 Global Medical Affairs, Almirall S.A., Barcelona, Spain; 3 O. Meany Consultancy GmbH, Hamburg, Germany; 4 Schmerz- und Palliativzentrum Göppingen, Göppingen, Germany

**Keywords:** Nabiximols, Oromucosal Spray, Long-Acting Opioid Analgesics, Neuropathic Pain, Low Back Pain, Real-World Data

## Abstract

**Objective:**

To compare the effectiveness, safety, and tolerability of add-on nabiximols (NBX) oromucosal spray vs typical oral long-acting opioid (LAO) analgesics in patients with severe (± chronic) peripheral neuropathic back pain poorly responsive to other treatments.

**Methods:**

Retrospective analysis of anonymized, propensity score–matched data from the German Pain e-Registry of adult outpatients who initiated NBX or LAO between March 2017 and March 2020.

**Results:**

Data were analyzed from propensity score–matched patients treated with NBX (n = 655) or LAO (n = 655): mean age ≈51 years; 57% female; mean pain duration ≈2.6 years; chronic pain 61%; severe dysfunctional pain 93%. At 6 months, NBX was noninferior to LAO for overall symptom relief, based on the least-squares mean difference between cohorts in change from baseline in patient-reported, pain-related aggregated nine-item scale scores (−27.84%; 95% confidence interval [CI] −29.71 to −25.96; *P* < 0.001) and individual pain-related scale scores. Subsequent prespecified superiority analysis of the primary endpoint showed that NBX was superior to LAO: all secondary endpoints measuring symptoms of pain and physical function improved significantly with NBX and LAO, with between-group differences favoring NBX (all *P* < 0.001). Fewer patients treated with NBX than LAO experienced treatment-related adverse events (25.5% vs 76.0%; *P* < 0.001) or discontinued treatment because of treatment-related adverse events (7.9% vs 29.3%; *P* < 0.001).

**Conclusion:**

Within study limitations (e.g., observational design, all potential biases), add-on NBX was superior to and better tolerated than add-on treatment with typical oral LAO analgesics in patients with neuropathic back pain inadequately controlled by recommended/established systemic therapies.

## Introduction

Chronic (low) back pain (LBP) is a common musculoskeletal disorder with an estimated global prevalence of 7.5% in 2017 [1, 2]. It has generally been considered a mixed pain syndrome with both neuropathic and nociceptive pain mechanisms, the latter being present in the majority of cases [1]. The neuropathic component of chronic LBP is often underrecognized and undertreated despite an acknowledged presence in 16–55% of cases [3–8]. Neuropathic pain originates from lesions within a degenerated disc (local neuropathic pain) after prolonged mechanical compression of the nerve root (mechanical neuropathic root pain) and/or from the effect of inflammatory mediators released by a degenerated disc on nerve fibers (inflammatory neuropathic root pain) [1, 9]. LBP with a neuropathic component is associated with more severe symptoms, an increased likelihood and severity of comorbidities (e.g., depression and anxiety), and higher health care costs [3, 7, 10].

Chronic LBP with a neuropathic component is difficult to treat and often refractory to the few available approved and recommended treatments. Anticonvulsants (e.g., pregabalin, gabapentin), antidepressants (e.g., amitriptyline, nortriptyline), and serotonin noradrenaline reuptake inhibitors are usual first-line treatment options for neuropathic pain [11], although robust data supporting their use in LBP are lacking [1]. Opioids alone have shown only moderate and short-term efficacy in managing neuropathic pain, and because of tolerability issues and risk of dependence, they are generally not recommended as first- or second-line therapy or for long-term treatment [1]. Nevertheless, opioid prescription for neuropathic LBP remains common in daily practice [12], underpinning the need to identify effective non-opioid options.

Cannabinoids have been shown in preclinical studies to exert analgesic effects by influencing inhibitory pathways and pathophysiological processes that play an important role in neuropathic pain [13, 14]. Among currently available cannabinoid-based medicines, Sativex^®^ (GW Pharmaceuticals, Cambridge, UK; United States Adopted Names [USAN]: nabiximols [NBX]) oromucosal spray containing approximately equal quantities of Δ9-tetrahydrocannabinol and cannabidiol, together with other cannabinoid and non-cannabinoid components [15], has undergone the most extensive scientific evaluation for neuropathic pain. Published randomized controlled trials (RCTs) of NBX in patients with neuropathic pain arising from conditions such as multiple sclerosis, spinal cord injury, allodynia, and brachial plexus injury suggested benefit in providing pain relief [16–20]. A recent meta-analysis of RCTs of add-on NBX for neuropathic pain reported significant benefit compared with placebo [21]. Some evidence also exists for benefit with inhaled cannabis in relieving neuropathic pain of varying origin [22].

NBX is produced from two chemovars of the *Cannabis sativa* plant, with each clone producing a high level of Δ9-tetrahydrocannabinol or cannabidiol [15]. The formulation is standardized to ensure quality, consistency, and stability [15]. In Europe and other territories, NBX oromucosal spray is indicated for symptom improvement in adult patients with moderate to severe spasticity due to multiple sclerosis who have not responded adequately to other antispasticity medication and who demonstrate clinically significant improvement in spasticity-related symptoms during an initial trial of therapy [23]. NBX has been approved with the same label since 2010 in the United Kingdom and currently in 15 European Union (EU) countries [23]. In Germany, since March 2017, physicians have been permitted to prescribe medical cannabis products for on- or off-label use in patients with severe disease resistant to available therapeutic options [24]. A published exploratory analysis of anonymized 12-week data from the German Pain e-Registry (GPeR) database for 800 adults treated in clinical practice with add-on NBX oromucosal spray for severe chronic pain refractory to other analgesics suggested that NBX oromucosal spray was effective in providing pain relief, particularly for neuropathic pain [12]. Conversely, RCTs of NBX in advanced cancer pain report mixed results [25–29].

The present study aimed to evaluate the comparative effectiveness of NBX oromucosal spray and typical oral long-acting opioid (LAO) analgesics (morphine, hydromorphone, oxycodone) administered as add-on therapy in patients with refractory severe (±chronic) peripheral neuropathic LBP under legislative conditions for use of cannabis as (pain) medicine in daily practice, enacted by the Parliament of the Federal Republic of Germany on March 10, 2017. The primary objective of the study was to compare the effectiveness of NBX oromucosal spray with that of LAO analgesics in comparable patient populations of the GPeR by evaluating a composite primary endpoint comprised of an aggregate of self-report pain measures. Secondary objectives were to evaluate the safety and tolerability of NBX oromucosal spray vs LAO analgesics by analyzing the nature and incidence of treatment-related adverse events (TRAEs) and treatment discontinuations due to TRAEs.

## Methods

### Study Design

This was a retrospective, open-label, parallel-group, flexible-dose, daily clinical practice study to compare the effectiveness, safety, and tolerability of add-on treatment with NBX oromucosal spray or typical oral LAO analgesics in adult patients with neuropathic back pain (NBP) who had insufficient pain relief in response to recommended/established systemic treatments. To homogenize samples, anonymized real-world data from the GPeR, which originally had been prospectively sampled for routine care purposes, were matched for relevant pathological features before analysis. The evaluation covered 13 time points: baseline and 12 post-baseline time points during treatment with between-assessment periods of 2 weeks. The maximum duration of treatment evaluation for a single patient was 6 months.

The GPeR is a national Web-based pain treatment registry involving more than 200 pain centers across the country. It was developed by the Institute of Neurological Sciences on behalf of the German Pain Association [30]. The GPeR serves as a standard e-tool to capture patient-reported data, and it fulfills physicians’ regulatory obligations according to the German Social Conduct of Law (V) for standardized documentation of patients under treatment for severe/chronic pain. The GPeR uses electronic case report forms to gather and evaluate patient-reported information relating to demography, pain characteristics, antecedents, pretreatment, and treatment response in daily practice. Under routine use of the GPeR, data are entered primarily by patients, then are checked by physicians or other engaged health care professionals, and are supplemented by related physician information where appropriate and required. After confirmation, the dataset for individual time points is locked and cannot be further changed. Patient questionnaires are those recommended by the German Pain Association, German Pain Society, and German Pain League for baseline and follow-up evaluations of patients suffering from chronic pain. The questionnaires incorporate a broad spectrum of validated instruments addressing various pain parameters, including pain chronification (Mainz Pain Staging System), pain severity (von Korff questionnaire), pain phenomenology, pain intensity, pain-related disabilities in daily life, quality of life, overall well-being, depression, anxiety and stress, treatment data, and TRAEs. TRAEs reported by patients are checked by physicians and confirmed or corrected with respect to their relationship with a specific treatment before the information entry is locked.

### Patient Selection and Study Cohorts

There was no formal sample size calculation for this analysis. Eligible for analysis were GPeR datasets of adult male or female outpatients with a pain history of ≥3 months, medically confirmed peripheral neuropathic LBP (as guided by a patient-reported painDETECT scale score ≥19 and substantiated by clinicians after input from patients, their medical history, and physical examination) with physician-confirmed inadequate pain relief after recommended first- or second-line treatments [11], who had received first administration of add-on NBX oromucosal spray or oral LAO analgesics between March 10, 2017, and March 31, 2020. Treatment initiation was defined as no study medication use in the previous 12 weeks. The date of the first dose was the index date for defining the 6-month evaluation period. Excluded from analysis were datasets of patients with active cancer and/or cancer-related pain; chemotherapy-induced neuropathic pain; HIV and/or HIV-related neuropathy; no follow-up evaluation after baseline documentation; or a diagnosis of osteoarthritis, rheumatoid arthritis, chronic widespread pain, complex regional pain syndrome, trigeminal autonomic cephalalgia, any painful lesions of the cranial nerves, or additional peripheral/central neuropathic pain problems.

Identified datasets encompassed two cohorts: patients who began treatment with NBX oromucosal spray and those who began treatment with oral LAO analgesics as add-on therapy to current underlying systemic analgesia. Propensity score matching (1:1), in which the nearest-neighbor method was used without replacement (caliper 0.15), was used to control for 11 predefined potential confounding factors: age, gender, average 24-h pain intensity index (PIX), pain severity, stage of chronification, duration of pain, comorbidities, comedications, indications/diagnosis for treatment, previous pain medication (taken and stopped before baseline), and current pain medication (background and rescue at onset of index medication). Propensity score matching is a statistical technique applied to observational data that attempts to estimate the effect of an intervention (treatment or other) by accounting for covariates that predict whether a patient receives the treatment, with the aim of reducing bias due to confounding variables [31].

### Study Medication

Because of the retrospective nature of this study, no formal dosing guidelines were in place for study medications. Treatment with NBX oromucosal spray or oral LAO analgesics for pain relief followed medical requirements according to the previous decision of participating physicians and was based exclusively on individual patient needs without any external specifications aside from recommendations in the current product information.

### Assessments

The primary efficacy variable was the relative change in the aggregated nine-item symptom relief (ASR-9) score over the 6-month evaluation period. The ASR-9 is a composite measure of nine distinct pain-related parameters assessed with validated patient-reported instruments aimed to broadly reflect patients’ pain evolution. Instruments include the average 24-h PIX reported on a 100-mm visual analog scale (VAS; 0 = no pain and 100 = worst pain conceivable), pain-related disabilities in daily life (modified pain disability index), overall physical and mental quality of life (12-item Short-Form Health Survey Physical Component Summary and Mental Component Summary), overall well-being (seven-item Marburg Questionnaire on Habitual Well-Being), pain-related depression/anxiety/stress (21-item Depression, Anxiety, and Stress Scales), and pain phenomenology (seven-item painDETECT Questionnaire). The ASR-9 score was calculated as the mean (percent) change relative to baseline in the nine individual measures assessed at weeks 2, 4, 6, 8, 12, 14, 16, 18, 20, 22, and 24 of the 6-month observation period. The ASR-9 score is reported on a scale ranging from deterioration (−100 = maximum aggravation) to no change (0) to improvement (+100 = maximum relief).

Secondary efficacy outcomes were relative (percent) change from baseline in each of the nine individual pain-related parameters of the ASR-9 instrument, and treatment response which was defined by usual response criteria in chronic pain studies as the proportion of patients with:

- Reduction in 24-h PIX of greater than or equal to the minimum clinically important difference (MCID) of 20 mm on the VAS.
- Reduction in 24-h PIX of greater than or equal to 50% from baseline.
- Reduction in 24-h PIX of greater than or equal to the tailored treatment target, which was defined by patients at baseline and before the onset of study treatment.

For ASR-9 components other than PIX, reported outcomes included the relative (percent) change from baseline and the proportions of patients with reductions greater than or equal to the MCID (for each parameter) and greater than or equal to 50% from baseline.

Safety was assessed by summarizing and analyzing the incidence and nature of TRAEs reported via the GPeR, the number of patients with TRAEs, and TRAE-related discontinuations. A TRAE was defined as an injury resulting from medical intervention possibly, probably, or definitely related to one of the drugs under evaluation that was newly reported or reported to worsen in severity after initiation of any treatment under evaluation during the evaluation period. Patient-reported TRAEs were checked and confirmed by physicians and transferred verbatim into Medical Dictionary for Regulatory Activities terms (MedDRA Version 23.0, March 2020), which were summarized by treatment cohort. The “serious adverse events” classification, as per clinical trial construct, does not apply for event reporting in daily practice by patients using the GPeR e-tool.

### Statistical Analyses

Primary and secondary efficacy analyses were conducted on data from the modified intent-to-treat population, which was defined as patients who had 1) documented intake of at least one dose of the treatments under evaluation and 2) at least one post-baseline/post-dose measure. Primary and secondary analyses were based on a mixed-model repeated-measures analysis of covariance, with adjustment for age, gender, pain severity, stage of chronification, history/duration of pain, comorbidity, concomitant medication, prior medication, and baseline values. When imputation of missing data was necessary, the baseline-observation-carried-forward and last-observation-carried-forward methods were used. For values missing not at random (e.g., as a consequence of a premature treatment discontinuation due to drug-related adverse events, death, or lack of efficacy), the baseline observation was carried forward to the corresponding endpoint evaluation for all parameters except the bowel function index, for which the last non-missing post-baseline observation was carried forward to the corresponding endpoint for evaluation. For all values missing at random, the last-observation-carried-forward procedure was used to impute missing parameters. This was a strategic consideration aimed at classifying a premature treatment discontinuation due to lack of efficacy or intolerable side effects as a treatment failure.

Continuous variables were summarized descriptively by number of patients (n), mean, standard deviation, 95% confidence interval (CI) of the mean, and median and range (minimum–maximum). For categorical and ordinal variables, data were summarized by number (n) and percentage (%). For between-group comparisons of 2 × 2 contingency tables with a dichotomous/binomial trait, McNemar’s test (with the Edwards correction) was applied. Pearson’s chi-squared tests were used for categorical variables with multinomial expressions. Between-group comparisons of continuous variables were applied depending on the data distribution: Paired-samples *t* tests were performed for normally distributed data, and Wilcoxon’s signed rank test was performed for non-normal distributions. Where appropriate, odds ratios and relative risks, with 95% CIs, and numbers needed to treat/harm were calculated.

Noninferiority between NBX oromucosal spray and oral LAO analgesics was established if the upper limit of the 95% CI for the treatment difference (NBX minus LAO) in the least-squares (LS) mean change from baseline in the ASR-9 score over 6 months (at weeks 2 to 24) did not exceed the prespecified +5.0% margin in the statistical analysis plan. This narrow margin was selected to prevent superinterpretation of minor differences. If noninferiority was demonstrated, a superiority analysis of the primary endpoint was to follow. Superiority was rejected if 1) the 95% CI for the primary endpoint measure of both treatment cohorts overlapped, and/or 2) the 95% CI of the LS mean difference for the primary efficacy variable between both treatment groups included “0,” and/or 3) the upper limit of the 95% CI was greater than −5.0, and/or 4) at least one of the ASR-9 parameters failed to meet any of the above criteria. With the use of a multidimensional approach, the superiority of NBX oromucosal spray was confirmed if 1) all the above efficacy criteria were fulfilled and 2) the incidence of treatment discontinuations due to TRAEs was significantly lower in the NBX cohort than in the LAO cohort.

For safety variables, statistical comparisons between treatments were conducted. For binomial data, such as the proportion of patients with TRAEs, McNemar’s test (with the Edwards correction) was used. For continuous data, such as number of TRAEs or number of patients with TRAEs per cohort, Student’s *t* test was performed.

All statistical tests were carried out with a two-sided significance level of 0.05. All comparisons, except those for the primary endpoint, were considered secondary and not adjusted for multiplicity. For the primary endpoint, the Bonferroni correction was used to counteract the problem of multiple comparisons.

With the use of the same described methodologies, a subgroup analysis was performed for all patients with an average baseline 24-h VAS score ≥50 mm in order to calculate and compare between-group differences for changes from baseline in mean PIX scores, reductions in 24-h PIX ≥20 mm VAS (MCID), and reductions in 24-h PIX ≥50% from baseline.

Analyses were conducted in PASW Statistics (Version 18.0; IBM Corp., Armonk, NY, USA). Tables and graphs were built in Microsoft Excel (MS Office 365, version 1911; Microsoft Corp., Redmond, WA, USA).

### Ethical Considerations

This noninterventional treatment evaluation was conducted in line with the principles of the Declaration of Helsinki, conformed to relevant national and regulatory requirements, and was approved by the ethics committees of the German Pain Association and German Pain League. Patients and physicians provided written informed consent before participation in the GPeR and agreed to the use of their anonymized data for health care research purposes. The study was registered in the electronic database of the European Medicine Agency for noninterventional studies (European Network of Centres for Pharmacoepidemiology and Pharmacovigilance [ENCEPP]: European Union Electronic Register of Post-Authorisation Studies [EU PAS Register] number EUPAS38969). All analyses were performed on anonymized data to comply with national guidelines on the protection of data privacy and the EU General Data Protection Regulation. Use of the electronic documentation platform iDocLive^®^ (O.Meany MD&PM GmbH, Nürnberg, Germany) and access to the GPeR was free of charge for members of the German Pain Association and for all patients, regardless of their insurance status.

## Results

### Study Population and Patient Disposition

After study selection criteria had been applied, 655 patients treated with NBX oromucosal spray and 655 propensity-matched patients treated with oral LAO analgesics were included in the analysis from pools of 827 and 8,779 NBX and LAO cases, respectively . By the end of the 6-month evaluation period, 192 patients (29.3%) treated with NBX and 301 patients (46.0%) treated with LAO analgesics had discontinued treatment (*P* < 0.001). Main reasons for treatment discontinuation were drug-related adverse events (7.9% vs 29.3%; *P* < 0.001), lack of efficacy (5.2% vs 5.5%), successful treatment (13.6% vs 8.5%), and no specific reason (2.6% vs 2.6%).

### Demographic and Baseline Characteristics

There were no clinically relevant differences between the NBX and LAO cohorts in terms of patient demographics, baseline clinical characteristics, and number and type of previous pain medications, including mild and strong opioids (Table 1). In each cohort, mean age was about 51 years, 57% were female, mean body mass index was 27.0 kg/m^2^, mean duration of pain was approximately 2.6 years, 61% had chronic pain (Mainz Pain Staging System stage III), and 93% had severe dysfunctional pain (von Korff grade 3 or 4). All patients had neuropathic pain, and the majority (86%) of patients in each cohort suffered from “other disorders of the back and spine” (*International Classification of Diseases* [ICD]-10 codes M50–54). There was a mean of 3.8 ± 1.92 comorbid conditions per patient, most commonly diseases of the circulatory system; endocrine, nutritional, and metabolic diseases; and diseases of connective tissue and the musculoskeletal system. Patients were taking a mean of 5.6 non-analgesic co-medications at baseline for comorbidities, most commonly drugs for the gastrointestinal tract and metabolism or for the cardiovascular system. Patients had been treated by a mean of 8.7 physicians and had received a mean of 7.9 previous pain medications. Tailored treatment targets chosen by patients at treatment onset and baseline scores for individual components of the ASR-9 were comparable between cohorts.

**Table 1.** Baseline characteristics

Characteristic	NBX (n = 655)	LAO (n = **655)**	NBX vs LAO (*P* Value)
Age, years, mean (SD)	51.4 (12.8)	51.2 (12.9)	0.770
Female, n (%)	373 (56.9)	373 (56.9)	1.000
Body mass index, kg/m^2^, mean (SD)	27.0 (4.9)	27.0 (4.9)	0.987
Stage of chronification, n (%)			
MPSS I	0 (0)	0 (0)	1.000
MPSS II	253 (38.6)	253 (38.6)	
MPSS III	402 (61.4)	402 (61.4)	
Chronic pain grade, n (%)			
von Korff 1	0 (0)	0 (0)	1.000
von Korff 2	43 (6.6)	43 (6.6)	
von Korff 3	235 (35.9)	235 (35.9)	
von Korff 4	377 (57.6)	377 (57.6)	
Type of NBP (ICD-10 classes M50–M54), n (%)			
Kyphosis and lordosis—M40	2 (0.3)	2 (0.3)	1.000
Scoliosis—M41	4 (0.6)	4 (0.6)	
Spinal osteochondrosis—M42	12 (1.8)	12 (1.8)	
Other deforming dorsopathies—M43	20 (3.1)	20 (3.1)	
Ankylosing spondylitis—M45	2 (0.3)	2 (0.3)	
Spondylosis—M47	38 (5.8)	38 (5.8)	
Other spondylopathies—M48	11 (1.7)	11 (1.7)	
Cervical disc disorders—M50	23 (3.5)	23 (3.5)	
Other disc disorders—M51	219 (33.4)	219 (33.4)	
Other dorsopathies, not elsewhere classified—M53	78 (11.9)	78 (11.9)	
Back pain—M54	246 (37.6)	246 (37.6)	
Pain duration, days, mean (SD)	952.1 (763.0)	958.6 (767.6)	0.897
Number of physicians involved, mean (SD)	8.70 (1.5)	8.70 (1.5)	0.678
Comorbidities, mean (SD)	3.78 (1.92)	3.78 (2.05)	0.978
No comorbidities, n (%)	20 (3.1)	12 (1.8)	0.020
Most common comorbidities (>30% of patients), n (%)			
Diseases of the circulatory system	395 (60.3)	398 (60.8)	0.810
Endocrine, nutritional, and metabolic diseases	303 (46.3)	301 (46.0)	0.875
Diseases of the musculoskeletal system and connective tissue	277 (42.3)	278 (42.4)	0.937
Diseases of the eye and adnexa	238 (36.3)	245 (37.4)	0.572
Diseases of the respiratory system	220 (33.6)	224 (34.2)	0.742
Diseases of the digestive system	212 (32.4)	209 (31.9)	0.801
Diseases of the ear and mastoid process	212 (32.4)	211 (32.2)	0.933
Number of non-analgesic co-medications at BL, mean (SD)	5.64 (3.17)	5.65 (2.99)	0.971
Most commonly affected systems, n			
Alimentary tract and metabolism	970	973	0.923
Cardiovascular system	820	818	0.944
Number of previous pain medications, mean (SD)	7.86 (2.42)	7.92 (2.25)	0.619
Patients with ≥10 previous pain medications, n (%)	159 (24.27)	159 (24.27)	0.619
Number of previous analgesics by type, mean (SD)			
Non-opioid analgesics	1.03 (0.81)	1.06 (0.84)	0.725
NSAIDs	1.29 (0.95)	1.31 (0.93)	0.791
Mild opioid analgesics*	1.20 (0.79)	1.12 (0.80)	0.077
Strong opioid analgesics^†^	1.16 (0.93)	1.17 (0.94)	0.906
Antidepressants	2.22 (1.24)	2.30 (1.30)	0.241
Anticonvulsants	0.95 (0.86)	0.95 (0.81)	0.947
Tailored treatment target (mm VAS), mean (SD)	27.3 (10.6)	27.3 (10.4)	0.985
Pain-related parameters (ASR-9 components)			
Average 24-h PIX (mm VAS), mean (SD)	43.3 (14.)	43.1 (14.0)	0.801
Pain-related disabilities (mPDI), mean (SD)	64.9 (17.8)	64.8 (17.3)	0.991
Physical quality of life (SF/VR12-PCS; NRS100), mean (SD)	31.0 (4.4)	31.0 (5.0)	0.976
Mental quality of life (SF/VR12-MCS; NRS100), mean (SD)	38.3 (8.8)	38.1 (10.2)	0.746
Overall well-being (MFHW; NRS5), mean (SD)	1.1 (0.5)	1.1 (1.1)	0.873
Depression (DASS-D; NRS21), mean (SD)	16.7 (3.7)	16.7 (3.6)	0.958
Anxiety (DASS-A; NRS21), mean (SD)	15.3 (4.0)	15.2 (3.8)	0.983
Stress (DASS-S; NRS21), mean (SD)	18.7 (2.3)	18.7 (2.3)	0.865
Quality of life impairment by pain (QLIP; NRS41), mean (SD)	16.7 (5.4)	16.7 (5.5)	0.915
Pain phenomenology (PDQ7; NRS35), mean (SD)	22.8 (3.6)	22.8 (3.2)	0.922

* Codeine, dihydrocodeine, tilidin ± naloxone, tramadol, and other.

† Morphine, hydromorphone, oxycodone ± naloxone, fentanyl, buprenorphine, tapentadol, other.

BL= baseline; DASS= Depression, Anxiety and Stress Scale; MFHW= Marburg Questionnaire on Habitual Well-Being; mPDI= modified pain disability index; MPSS= Mainz Pain Staging System; NRS= numerical rating scale; NSAIDs= nonsteroidal anti-inflammatory drugs; PDQ7 = painDETECT Questionnaire; QLIP= quality of life impairment by pain inventory; SD= standard deviation; SF-12 MCS= 12-item Short-Form Health Survey Mental Component Summary; SF-12 PCS= 12-item Short-Form Health Survey Physical Component Summary.

At baseline, patients were taking an average of 3.7 background pain medications, most frequently antidepressants (mean of 1.8 medications per patient) (Table 2). More than half of patients in each group (NBX: 56.2%; LOA: 55.4%) required rescue pain medication at baseline (mean of 0.76 medications per patient in both groups), most commonly strong opioid analgesics (mean of 0.43 strong opioid analgesics per patient and 0.42 strong opioid analgesics per patient, respectively).

**Table 2.** Concomitant pain medications at baseline and month 6 in patients treated with NBX or oral LAO analgesics

Type	At Baseline Mean (SD)			At End of Month 6 Mean (SD)		
	NBX	LAO	NBX vs LAO (*P* Value)	NBX	LAO	NBX vs LAO (*P* Value)
	(n = 655)	(n = 655)		(n = 655)	(n = 655)	
**Background pain medication**						
Patients, n (%)	655 (100)	655 (100)	1.000	238 (36.3)	418 (63.8)	<0.001
Number of background pain medications, mean per patient (SD)	3.67 (1.56)	3.66 (1.49)	0.928	1.04 (1.68)	1.93 (1.92)	<0.001
Number of background pain medications by type, mean per patient (SD)						
Non-opioid analgesics	0.20 (0.42)	0.19 (0.41)	0.842	0.03 (0.19)	0.07 (0.26)	<0.001
NSAIDs	0.13 (0.34)	0.13 (0.33)	0.804	0.01 (0.11)	0.05 (0.22)	<0.001
Mild opioid analgesics*	0.19 (0.40)	0.19 (0.41)	0.946	0.02 (0.14)	0.07 (0.27)	<0.001
Strong opioid analgesics^†^	0.55 (0.85)	0.54 (0.83)	0.895	0.22 (0.59)	0.21 (0.60)	0.926
Antidepressants	1.76 (1.03)	1.78 (1.05)	0.750	0.43 (0.90)	1.18 (1.22)	<0.001
Anticonvulsants	0.84 (0.65)	0.83 (0.64)	0.731	0.33 (0.59)	0.33 (0.57)	1.000
Patients who stopped ≥1 background medication, n (%)				520 (79.4)	374 (57.1)	<0.001
NBX vs LAO OR (95% CI)				2.894 (2.266–3.696)		
NBX vs LAO RR (95% CI)				1.390 (1.287–1.501)		
NNT				4		
**Rescue pain medication**						
Patients, n (%)	368 (56.2)	363 (55.4)	0.694	54 (8.2)	224 (34.2)	<0.001
Number of rescue pain medications, mean per patient (SD)	0.76 (0.81)	0.76 (0.81)	0.946	0.11 (0.41)	0.47 (0.74)	<0.001
Number of rescue pain medications by type, mean per patient (SD)						
Non-opioid analgesics	0.18 (0.39)	0.18 (0.39)	0.944	0.03 (0.16)	0.10 (0.30)	<0.001
NSAIDs	0.09 (0.30)	0.10 (0.31)	0.929	0.02 (0.15)	0.05 (0.23)	0.002
Mild opioid analgesics*	0.06 (0.50)	0.06 (0.24)	1.000	0.00 (0.06)	0.04 (0.20)	<0.001
Strong opioid analgesics^†^	0.43 (0.61)	0.42 (0.59)	0.854	0.06 (0.27)	0.28 (0.52)	<0.001
Patients who stopped ≥1 rescue medication, n (%)				314 (47.9)	139 (21.2)	<0.001
NBX vs LAO OR (95% CI)				3.418 (2.683–4.354)		
NBX vs LAO RR (95% CI)				2.259 (1.91–2.672)		
NNT				4		

* Codeine, dihydrocodeine, tilidin ± naloxone, tramadol, and other.

† Morphine, hydromorphone, oxycodone ± naloxone, fentanyl, buprenorphine, tapentadol, other.

NNT= number needed to treat; NSAIDs= nonsteroidal anti-inflammatory drugs; OR= odds ratio; RR= relative risk; SD= standard deviation.

### Study Medication

Patients in the LAO group had initiated add-on treatment with oxycodone (± naloxone; n = 302; 46.1% of sample), hydromorphone (n = 192, 29.3%), and morphine (n = 161, 24.6%).

Over the 6-month observation period, the mean dose of NBX oromucosal spray was 6.2 ± 2.4 sprays/day, and the mean morphine-equivalent dose of LAO analgesics was 69.4 ± 38.9 mg/day, including the uptitration period in both treatment groups. At weeks 4, 6, and 8, the reported NBX mean daily dose was 3.8 ± 1.9, 5.1 ± 1.8, and 6.1 ± 1.6 sprays/day, respectively. From week 12 onward, patients taking NBX reported a stable mean dose of 7.1 ± 2.1 sprays/day. Patients taking LAO analgesics had a longer dose titration period, reaching a stable mean morphine-equivalent dose of 84.4 mg/day at week 20.

### Treatment Duration

Mean treatment duration was significantly longer in patients treated with NBX than in patients treated with LAO (136.2 ± 29.9 vs 118.3 ± 53.0 days; *P* < 0.001), and median treatment duration was similar (165 vs 163 days). Among patients who discontinued treatment prematurely, mean treatment duration was 58.4 ± 29.9 (range 6–148) days in the NBX cohort and 68.0 ± 53.0 (range 2–175) days (*P* = 0.969) in the LAO cohort. Corresponding values among patients who did not discontinue treatment prematurely were 168.0 ± 4.2 (range 161–175) days and 167.9 ± 4.4 (range 161–175) days, respectively (*P* = 0.750).

### Concomitant Pain Medication

Use of concomitant background pain medication (frequency and type) was comparable between the groups at baseline (Table 2). After 6 months’ treatment, the number of background pain medications used per patient was significantly reduced in the NBX cohort (from 3.67 at baseline to 1.04) and in the LAO cohort (from 3.66 to 1.93), with the reduction favoring NBX (*P* < 0.001 vs LAO). At 6 months, significantly fewer patients treated with NBX vs LAO were using background pain medications (36.3% vs 63.8%; *P* < 0.001), and significantly more NBX-treated patients than LAO-treated patients had stopped taking at least one other pain medication (79.4% vs 57.1%; *P* < 0.001). The frequency and type of concomitant non-pain medications showed no relevant differences between the cohorts at week 24 (*P* = 0.978; data not shown).

Use of rescue pain medication (frequency and type) was comparable between the groups at baseline (Table 2). The number of rescue medications used per patient was reduced in the NBX cohort (from 0.76 at baseline to 0.11 at 6 months) and in the LAO cohort (from 0.76 at baseline to 0.47 at 6 months), with the difference favoring NBX (*P* < 0.001 vs LAO). The proportion of patients using rescue medications decreased with both NBX oromucosal spray (56.2% at baseline to 8.2% at month 6; *P* < 0.001) and LAO analgesics (55.4% to 34.2%; *P* < 0.001), with the magnitude of difference favoring NBX (*P* < 0.001 vs LAO). Significantly more patients treated with NBX than with LAO stopped taking ≥1 rescue pain medication (47.9% vs 21.2%; *P* < 0.001).

### Primary Endpoint

**Figure 1.**
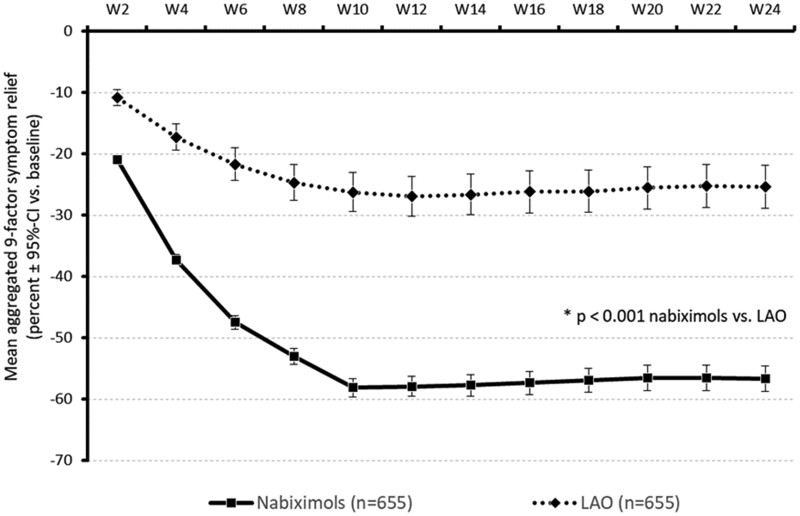
Mean relative (percent vs baseline) improvement in the ASR-9 score over time in patients treated with NBX (n = 655) or oral LAO analgesics (n = 655). *P < 0.001 vs oral LAO.

Treatment with NBX oromucosal spray or oral LAO analgesics for 6 months provided significant symptom relief in patients with NBP, as indicated by significant reductions from baseline in ASR-9 scores in both cohorts over weeks 2–24 (mean relative change −51.39 ± 24.0% and −23.55 ± 19.4%, respectively; both *P* < 0.001) (Table 3). The LS mean difference between cohorts was −27.84 (95% CI −29.71 to −25.96), confirming the noninferiority of NBX oromucosal spray (*P* < 0.001). A subsequent pre-established superiority analysis of the primary endpoint showed that NBX oromucosal spray was superior to LAO in providing symptom relief (Table 3). At each 2-week assessment time point, ASR-9 scores were significantly lower with NBX than with LAO (Figure 1).

**Table 3.** Noninferiority/superiority of NBX vs oral LAO analgesics

Noninferiority and/or Superiority Parameters	NBX (n = 655)	LAO (n = 655)	NBX vs LAO (*P* Value)
ASR-9			
Relative change, %, W2–24 vs BL, mean (SE)	−51.39 (24.0)	−23.55 (19.4)	<0.001
95% CI	−52.7 to −50.1	−24.9 to −22.2	
W2–24 LS mean diff. [NBX−LAO] (95% CI)	−27.84 (−29.71 to −25.96)		<0.001
PIX			
W2–24 (95% CI)	−71.46 to −67.13	−52.03 to −47.7	<0.001
W2–24 LS mean diff. [NBX−LAO] (95% CI)	−19.43 (−22.5 to −16.37)		
mPDI			
W2–24 (95% CI)	−67.78 to −64.01	−45.23 to −41.46	<0.001
W2–24 LS mean diff. [NBX−LAO] (95% CI)	−22.55 (−25.22 to −19.88)		
SF-12			
SF-12 PCS W2–24 (95% CI)	43.2 to 47.18	19.69 to 23.68	<0.001
SF-12 PCS W2–24 LS mean diff. [NBX−LAO] (95% CI)	23.51 (20.69 to 26.32)		
SF-12 MCS W2–24 (95% CI)	32.26 to 35.70	8.18 to 11.63	<0.001
SF-12 MCS W2–24 LS mean diff. [NBX−LAO] (95% CI)	24.08 (21.64 to 26.51)		
MFHW			
MFHW W2–24 (95% CI)	46.24 to 50.46	−5.75 to −1.53	<0.001
MFHW-MCS W2–24 LS mean diff. [NBX−LAO] (95% CI)	51.99 (49.00 to 54.98)		
DASS-D			
W2–24 (95% CI)	−51.44 to −47.97	−27.79 to −24.31	<0.001
W2–24 LS mean diff. [NBX−LAO] (95% CI)	−23.66 (−26.12 to −21.2)		
DASS-A			
W2–24 (95% CI)	−59.31 to −55.73	−28.7 to −25.13	<0.001
W2–24 LS mean diff. [NBX−LAO] (95% CI)	−30.61 (−33.13 to −28.08)		
DASS-S			
W2–24 (95% CI)	−63.64 to −60.6	−30.4 to −27.36	<0.001
W2–24 LS mean diff. [NBX−LAO] (95% CI)	−33.23 (−35.38 to −31.08)		
PDQ7			
W2–24 (95% CI)	−32.52 to −28.42	−10.98 to −6.87	<0.001
W2–24 LS mean diff. [NBX−LAO] (95% CI)	−21.55 (−24.45 to − 18.64)		
TRAE-related treatment discontinuations, n (%)	52 (5.9)	192 (14.8)	<0.001

BL= baseline; DASS= Depression, Anxiety, and Stress Scales; MFHW= Marburg Questionnaire on Habitual Well-Being; mPDI= modified pain disability index; PDQ7 = painDETECT Questionnaire; QLIP= quality of life impairment by pain inventory; SE= standard error; SF-12 MCS= 12-item Short-Form Health Survey Mental Component Summary; SF-12 PCS= 12-item Short-Form Health Survey Physical Component Summary; W2–24 = weeks 2 to 24.

### Secondary Endpoints

**Figure 2.**
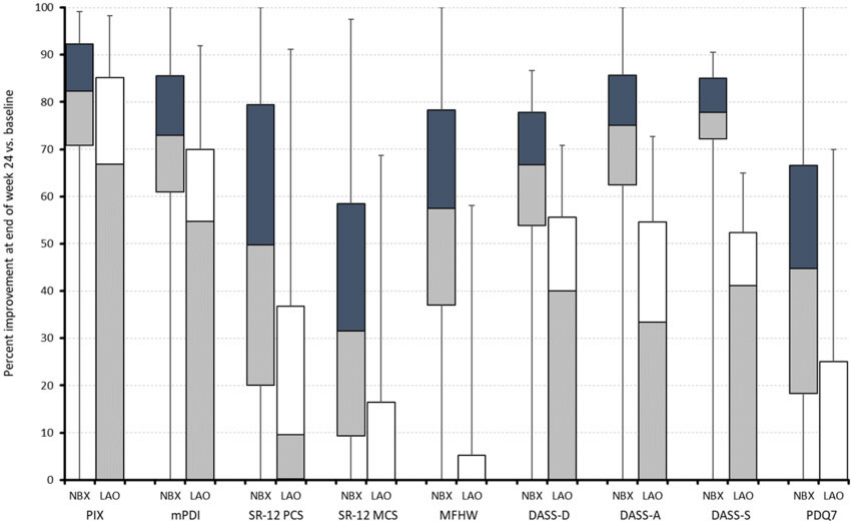
Relative improvement (%) from baseline in individual components of the ASR-9 in patients treated with NBX (n = 655) or oral LAO analgesics (n = 655). Box plots show median (middle horizontal line in the box), 25% and 75% quartiles (bottom and top lines of the box), and whiskers corresponding to the 2.5–97.5% quartiles. Blue upper boxes: relative improvement with NBX. White upper boxes: relative improvement with LAO. DASS= Depression, Anxiety, and Stress Scale; MFHW= Marburg Questionnaire on Habitual Well-Being; mPDI= modified pain disability index; PDQ7 = painDETECT Questionnaire; SF-12 MCS= 12-item Short-Form Health Survey Mental Component Summary; SF-12 PCS= 12-item Short-Form Health Survey Physical Component Summary.

Mean relative changes (%) from baseline for individual components of the ASR-9 in patients treated with NBX oromucosal spray or oral LAO analgesics are shown in Figure 2. For all components, relative changes from baseline were significantly greater for NBX than for LAO (all *P* < 0.001).

#### Pain Parameters

At 6 months, scores relative to baseline were significantly improved with NBX oromucosal spray and LAO analgesics for lowest 24-h pain intensity (mean relative change −70.9% vs −47.1%; *P* < 0.001), medium 24-h pain intensity (−68.2% vs −47.9%; *P* < 0.001), highest 24-h pain intensity (−75.2% vs −50.4%; *P* < 0.001), and 24-h average pain intensity (−72.3% vs −49.2%; *P* < 0.001), with between-group differences significantly in favor of NBX over LAO (all *P* < 0.001) (Table 4). Significantly more patients treated with NBX oromucosal spray than with LAO documented PIX improvement greater than or equal to the MCID (79.8% vs 56.2%), ≥50% from baseline (86.1% vs 62.1%), and greater than or equal to the tailored treatment target recorded by patients before treatment onset (86.0% vs 63.1%), with corresponding odds ratios of 3.09–3.77, relative risks of 1.36–1.42, and numbers needed to treat of 4 (all *P* < 0.001) (Table 4).

**Table 4.** Pain parameters in patients treated with NBX and oral LAO analgesics

Pain Parameters	NBX	LAO	NBX vs LAO
	(n = 655)	(n = 655)	(*P* Value)
Lowest 24-h pain intensity (mm VAS), mean (SD)			
BL	14.1 (15.6)	14.0 (15.4)	0.979
Month 6	4.1 (9.3)	7.4 (12.9)	<0.001
Absolute change from BL	−10.0 (14.2)	−6.6 (13.9)	<0.001
Relative change (%) from BL	−70.9 (53.2)	−47.1 (53.6)	<0.001
*P* value vs BL	<0.001	<0.001	
Medium 24-h pain intensity (mm VAS), mean (SD)			
BL	44.7 (18.9)	44.5 (19.1)	0.855
Month 6	14.2 (16.4)	23.2 (22.6)	<0.001
Absolute change from BL	−30.5 (20.7)	−21.3 (21.6)	<0.001
Relative change (%) from BL	−68.2 (35.4)	−47.9 (42.6)	<0.001
*P* value vs BL	<0.001	<0.001	
Highest 24-h pain intensity (mm VAS), mean (SD)			
BL	71.1 (21.7)	70.7 (20.3)	0.749
Month 6	17.6 (24.0)	35.1 (31.4)	<0.001
Absolute change from BL	−53.5 (28.2)	−35.6 (30.7)	<0.001
Relative change (%) change from BL	−75.2 (32.4)	−50.4 (40.1)	<0.001
*P* value vs BL	<0.001	<0.001	
PIX (mm VAS), mean (SD)			
BL	43.3 (14.0)	43.1 (14.0)	0.801
Month 6	12.0 (14.7)	21.9 (19.2)	<0.001
Absolute change from BL	−31.3 (16.8)	−21.2 (18.5)	<0.001
Relative change (%) from BL	−72.3 (30.5)	−49.2 (39.9)	<0.001
*P* value vs BL	<0.001	<0.001	
PIX improvement from BL of greater than or equal to the MCID, n (%)	523 (79.8)	368 (56.2)	<0.001
NBX vs LAO OR (95% CI)	3.090 (2.417–3.950)		
NBX vs LAO RR (95% CI)	1.421 (1.315–1.536)		
NNT	4		
PIX improvement from BL of ≥50%, n (%)	564 (86.1)	407 (62.1)	<0.001
NBX vs LAO OR (95% CI)	3.777 (2.878–4.957)		
NBX vs LAO RR (95% CI)	1.386 (1.296–1.482)		
NNT	4		
PIX improvement from BL of greater than or equal to the TTT, n (%)	563 (86.0)	413 (63.1)	<0.001
NBX vs LAO OR (95% CI)	3.586 (2.733–4.705)		
NBX vs LAO RR (95% CI)	1.363 (1.276–1.456)		
NNT	4		

BL= baseline; NNT= number needed to treat; OR= odds ratio; RR= relative risk; TTT= tailored treatment target.

#### Health-Related Quality-of-Life Parameters

After 6 months’ treatment, all health-related quality-of-life parameters were improved significantly with NBX oromucosal spray and oral LAO analgesics, as indicated by mean relative changes from baseline in modified pain disability index scores (−66.1% and −42.9%), 12-item Short-Form Health Survey Physical Component Summary scores (49.4% and 21.3%), 12-item Short-Form Health Survey Mental Component Summary scores (33.9% and 9.7%), Marburg Questionnaire on Habitual Well-Being scores (mean 54.4% and 0.1%), Depression, Anxiety, and Stress Scales—Depression scores (mean −59.3% and −31.7%), Depression, Anxiety, and Stress Scales—Anxiety scores (−66.0% and −31.6%), Depression, Anxiety, and Stress Scales—Stress scores (−69.0% and −32.1%), and painDETECT Questionnaire scores (−34.6% and −10.1%) (Table 5). Improvements from baseline in all parameters were significantly greater in patients treated with NBX than in those treated with LAO (all *P* ≤ 0.001), with the greatest improvement observed in overall well-being (Marburg Questionnaire on Habitual Well-Being scores) relative to LAO (LS mean difference 51.99%; 95% CI 49–54.98%) (Table 3). For all parameters, significantly greater proportions of patients treated with NBX than with LAO had reductions from baseline of greater than or equal to the MCID and ≥50%, which corresponded to odds ratios of 3.37–15.46, relative risks of 1.39–6.98, and numbers needed to treat ranging from 2 to 5 (Table 5).

**Table 5.** Quality-of-life measures at month 6 in patients treated with NBX vs oral LAO analgesics

Measures	NBX (n = 655)	LAO (n = 655)	NBX vs LAO (*P* Value)
*Pain-related disabilities of daily life activities*			
Relative mPDI change (%) from BL, mean (SD)	−66.1 (28.7)	−42.9 (34.5)	<0.001
*P* value vs BL	<0.001	<0.001	
mPDI improvement vs BL ≥ MCID, n (%)	553 (84.4)	390 (59.5)	<0.001
NBX vs LAO OR (95% CI)	3.684 (2.833–4.79)		
NBX vs LAO RR (95% CI)	1.418 (1.321–1.523)		
NNT	4		
mPDI improvement vs BL ≥ 50%, n (%)	562 (85.8)	383 (58.5)	<0.001
NBX vs LAO OR (95% CI)	4.292 (3.28–5.616)		
NBX vs LAO RR (95% CI)	1.467 (1.366–1.576)		
NNT	4		
*Physical quality of life*			
Relative SF-12 PCS change (%) from BL, mean (SD)	49.4 (39.4)	21.3 (30.9)	<0.001
*P* value vs BL	<0.001	<0.001	
SF-12 PCS improvement vs BL ≥ MCID, n (%)	513 (78.3)	313 (47.8)	<0.001
NBX vs LAO OR (95% CI)	3.947 (3.102–5.022)		
NBX vs LAO RR (95% CI)	1.639 (1.499–1.793)		
NNT	3		
SF-12 PCS improvement vs BL ≥50%, n (%)	327 (49.9)	113(17.3)	<0.001
NBX vs LAO OR (95% CI)	4.782 (3.709–6.165)		
NBX vs LAO RR (95% CI)	2.894 (2.407–3.48)		
NNT	3		
*Mental quality of life*			
Relative SF-12 MCS change (%) from BL, mean (SD)	33.9 (31.9)	9.7 (24.7)	0.000
*P* value vs BL	<0.001	<0.001	
SF-12 MCS improvement vs BL ≥ MCID, n (%)	499 (76.2)	224 (34.2)	<0.001
NBX vs LAO OR (95% CI)	6.155 (4.834–7.837)		
NBX vs LAO RR (95% CI)	2.228 (1.987–2.498)		
NNT	2		
SF-12 MCS improvement vs BL ≥50%, n (%)	204 (31.1)	60 (9.2)	<0.001
NBX vs LAO OR (95% CI)	4.486 (3.281–6.133)		
NBX vs LAO RR (95% CI)	3.400 (2.604–4.439)		
NNT	5		
*Overall well-being*			
Relative MFHW change (%) from BL, mean (SD)	54.4 (32.4)	0.1 (35.7)	<0.001
*P* value vs BL	<0.001	<0.001	
MFHW improvement vs BL ≥ MCID, n (%)	553 (84.4)	180 (27.5)	<0.001
NBX vs LAO OR (95% CI)	14.307 (10.899–18.781)		
NBX vs LAO RR (95% CI)	3.072 (2.701–3.494)		
NNT	2		
MFHW improvement vs BL ≥50%, n (%)	384 (58.6)	55(8.4)	<0.001
NBX vs LAO OR (95% CI)	15.458 (11.26–21.222)		
NBX vs LAO RR (95% CI)	6.982 (5.378–9.064)		
NNT	2		
*Depression*			
Relative DASS-D change (%) from BL, mean (SD)	−59.3 (28.8)	−31.7 (32.0)	<0.001
*P* value vs BL	<0.001	<0.001	
DASS-D improvement vs BL ≥ MCID, n (%)	554 (84.6)	398 (60.8)	<0.001
NBX vs LAO OR (95% CI)	3.542 (2.721–4.611)		
NBX vs LAO RR (95% CI)	1.392 (1.298–1.492)		
NNT	4		
DASS-D improvement vs BL ≥50%, n (%)	516 (78.8)	232 (35.4)	<0.001
NBX vs LAO OR (95% CI)	6.768 (5.29–8.659)		
NBX vs LAO RR (95% CI)	2.224 (1.991–2.485)		
NNT	2		
*Anxiety*			
Relative DASS-A change (%) from BL, mean (SD)	−66.0 (31.3)	−31.6 (33.4)	<0.001
*P* value vs BL	<0.001	<0.001	
DASS-A improvement vs BL ≥ MCID, n (%)	567 (86.6)	397 (60.6)	<0.001
NBX vs LAO OR (95% CI)	4.187 (3.184–5.506)		
NBX vs LAO RR (95% CI)	1.428 (1.333–1.53)		
NNT	4		
DASS-A improvement vs BL ≥50%, n (%)	545 (83.2)	222 (33.9)	<0.001
NBX vs LAO OR (95% CI)	9.664 (7.444–12.547)		
NBX vs LAO RR (95% CI)	2.455 (2.194–2.747)		
NNT	2		
*Stress*			
Relative DASS-S change (%) from BL, mean (SD)	−69.0 (28.2)	−32.1 (25.1)	<0.001
*P* value vs BL	<0.001	<0.001	
DASS-S improvement vs BL ≥ MCID, n (%)	569 (86.9)	417 (63.7)	<0.001
NBX vs LAO OR (95% CI)	3.776 (2.862–4.982)		
NBX vs LAO RR (95% CI)	1.365 (1.279–1.457)		
NNT	4		
DASS-S improvement vs BL ≥ 50%, n (%)	556 (84.9)	204 (31.1)	<0.001
NBX vs LAO OR (95% CI)	12.416 (9.475–16.269)		
NBX vs LAO RR (95% CI)	2.725 (2.421–3.067)		
NNT	2		
*Pain phenomenology*			
Relative PDQ7 change (%) from BL, mean (SD)	−34.6 (31.9)	−10.1 (31.2)	<0.001
*P* value vs BL	<0.001	<0.001	
PDQ7 improvement vs BL ≥ MCID, n (%)	382 (58.3)	147 (22.4)	<0.001
NBX vs LAO OR (95% CI)	4.836 (3.802–6.151)		
NBX vs LAO RR (95% CI)	2.599 (2.223–3.039)		
NNT	3		
PDQ7 improvement vs BL ≥50%, n (%)	225 (34.4)	88 (13.4)	<0.001
NBX vs LAO OR (95% CI)	3.371 (2.557–4.445)		
NBX vs LAO RR (95% CI)	2.557 (2.049–3.191)		
NNT	5		

BL= baseline; DASS= Depression, Anxiety and Stress Scale; MFHW= Marburg Questionnaire on Habitual Well-Being; mPDI= modified pain disability index; NNT= number need to treat; OR= odds ratio; PDQ7 = painDETECT Questionnaire; RR= relative risk; SF-12 MCS= 12-item Short-Form Health Survey Mental Component Summary; SF-12 PCS= 12-item Short-Form Health Survey Physical Component Summary.

### Effect Sizes

**Figure 3.**
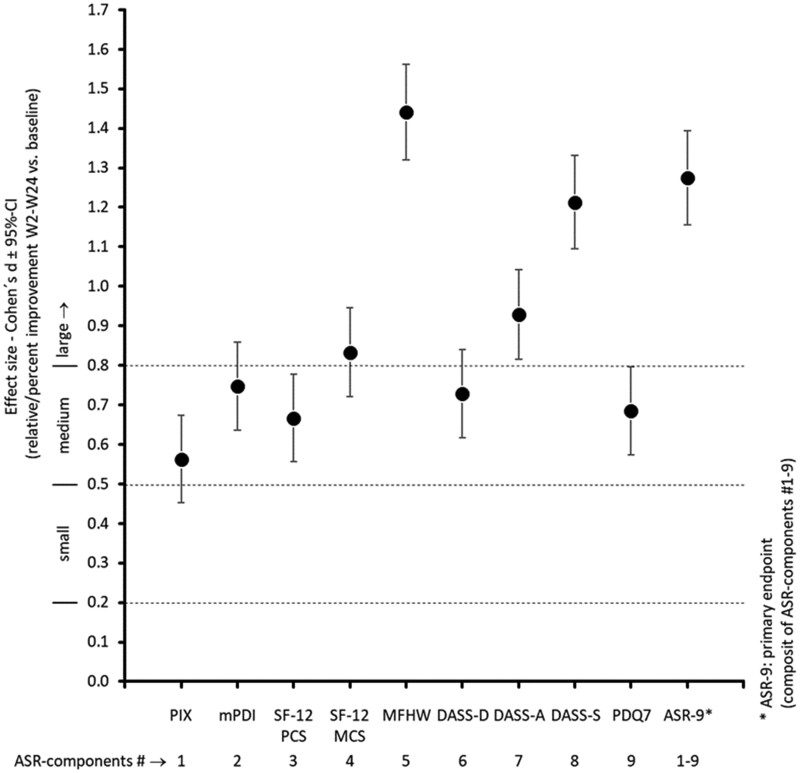
Effect sizes (Cohen’s *d*; 95% CIs) for individual components of the ASR-9 and composite ASR-9 score. Cohen’s *d* effect size legend: <0.2, insignificant; 0.2–0.5, small; 0.5–0.8, medium; >0.8, large. DASS= Depression, Anxiety, and Stress Scale; MFHW= Marburg Questionnaire on Habitual Well-Being; mPDI= modified pain disability index; PDQ7 = painDETECT Questionnaire; SF-12 MCS= 12-item Short-Form Health Survey Mental Component Summary; SF-12 PCS= 12-item Short-Form Health Survey Physical Component Summary.

Effect sizes (Cohen’s *d*; 95% CIs) of between-group differences for individual components of the ASR-9 and composite ASR-9 score are shown in Figure 3. For all measures, effect sizes in favor of NBX were biometrically significant and clinically relevant (all >0.5).

### Safety Endpoints

**Figure 4.**
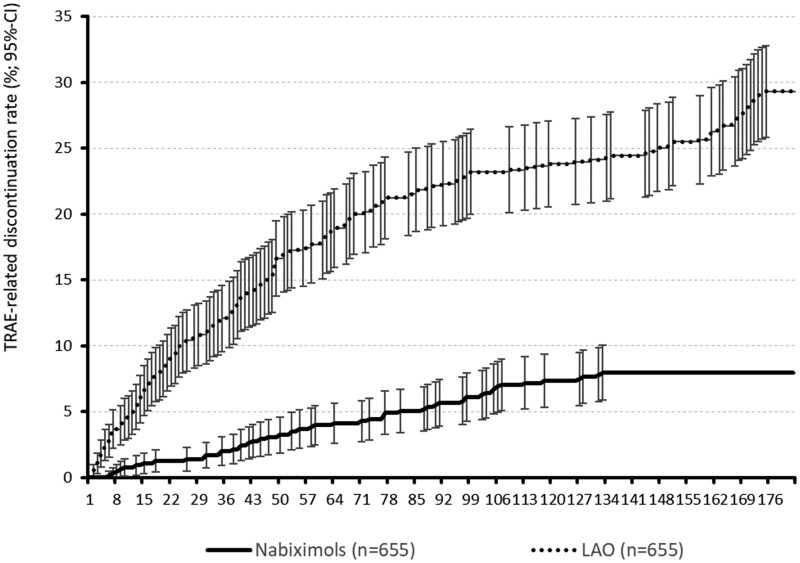
TRAE-related discontinuation profiles (rate ± 95% CI) for NBX and oral LAO analgesics during the 24-week evaluation period.

Over the 6-month observation period, significantly fewer TRAEs were reported by patients treated with NBX oromucosal spray than by patients treated with oral LAO analgesics (231 vs 861 TRAEs; *P* < 0.001; Table 6). Proportions of patients experiencing ≥1 TRAE (25.5% vs 76.0%; *P* < 0.001) or ≥2 TRAEs (7.0% vs 38.9%) were significantly lower in the NBX cohort vs the LAO cohort. Significantly fewer patients treated with NBX than with LAO discontinued treatment because of TRAEs (7.9% vs 29.3%; *P* < 0.001; Figure 4).

**Table 6.** Safety outcomes in patients treated with NBX or oral LAO analgesics

Safety Outcomes	NBX (n = 655)	LAO (n = 655)	NBX vs LAO (*P* Value)	LOA vs NBX OR (95% CI)	LOA vs NBX RR (95% CI)	NNH
Number of TRAEs recorded, n	231	861	<0.001			
Patients with ≥1 TRAE, n (%)	167 (25.5)	498 (76.0)	<0.001	9.269 (7.211–11.915)	2.982 (2.598–3.423)	2*
Patients with ≥2 TRAE, n (%)	46 (7.0)	255 (38.9)	<0.001	8.440 (6.017–11.838)	5.543 (4.128–7.443)	3*
Treatment discontinuations due to TRAEs, n (%)	52 (7.9)	192 (29.3)	<0.001	4.809 (3.459–6.686)	3.692 (2.772–4.917)	5*
TRAEs according to System Organ Class						
Gastrointestinal disorders, n (%)	71 (10.8)	351 (53.6)	<0.001	9.497 (7.104–12.696)	4.944 (3.925–6.228)	2
Nervous system disorders, n (%)	64 (9.8)	146 (22.3)	0.000	2.649 (1.93–3.636)	2.281 (1.736–2.997)	8
Psychiatric disorders, n (%)	28 (4.3)	105 (16.0)	0.000	4.275 (2.774–6.587)	3.750 (2.507–5.609)	9
Metabolism and nutrition disorders, n (%)	16 (2.4)	6 (0.9)	0.000	0.369 (0.143–0.949)	0.375 (0.148–0.952)	66
General disorders and administration site conditions, n (%)	14 (2.1)	60 (9.2)	<0.001	4.617 (2.553–8.349)	4.286 (2.42–7.591)	14
Musculoskeletal and connective tissue disorder, n (%)	12 (1.8)	26 (4.0)	0.000	2.215 (1.108–4.428)	2.167 (1.103–4.258)	47
Cardiac disorders, n (%)	8 (1.2)	5 (0.8)	0.245	0.622 (0.202–1.911)	0.625 (0.206–1.9)	218
Injury, poisoning, and procedural complications, n (%)	4 (0.6)	36 (5.5)	<0.001	9.465 (3.349–26.748)	9.000 (3.222–25.142	20
Vascular disorders, n (%)	2 (0.3)	1 (0.2)	0.000	0.499 (0.045–5.517)	0.500 (0.045–5.501)	655
Skin and subcutaneous tissue disorders, n (%)	1 (0.2)	9 (1.4)	0.000	9.111 (1.151–72.122)	9.000 (1.143–70.839)	82

NNH= number needed to harm; OR= odds ratio; RR= risk ratio.

* Number needed to treat.

In both cohorts, the most common TRAEs were gastrointestinal disorders (10.8% vs 53.6%), nervous system disorders (9.8% vs 22.3%), and psychiatric disorders (4.3% vs 16.0%), all occurring significantly less frequently in patients treated with NBX than in those treated with LAO (all *P* ≤ 0.001; Table 6). Metabolism and nutritional disorders occurred more frequently in the NBX cohort, although the incidence was low (2.4% vs 0.9% in the LAO cohort; *P* = 0.000). The incidence of cardiac disorders did not differ significantly between the two cohorts (1.2% vs 0.8%; *P* = 0.245). All other TRAEs occurred less frequently in patients treated with NBX than in those treated with LAO (Table 6).

### Subgroup Analyses

The subgroup analysis for patients with a baseline 24-h average pain intensity VAS score of ≥50 mm (n = 259 from the NBX group and n = 279 from the LAO group, corresponding to 39.5% and 42.6% of the overall sample, respectively) yielded results in favor of NBX. At 12 weeks, mean (standard deviation ) VAS PIX score improvements from baseline were from 63.3 (10.3) to 17.4 (18.5) in the NBX group (*P* < 0.001) and from 62.3 (10.1) to 30.6 (24.9) in the LAO group (*P* < 0.001), with the between-group difference significantly in favor of NBX (*P* < 0.001). Corresponding improvements at 24 weeks were from 63.3 (10.3) to 19.7 (20.7) in the NBX group (*P* < 0.001) and from 62.3 (10.1) to 33.4 (25.7) in the LAO group (*P* < 0.001), with the between-group difference significantly in favor of NBX (*P* < 0.001). At week 24, more patients treated with NBX than with LAO documented VAS reductions ≥20 mm MCID (85.7% vs 57.7%; *P* < 0.001) or ≥50% from baseline (82.6% vs 50.9%; *P* < 0.001).

## Discussion

This study compared the relative effectiveness and tolerability of NBX oromucosal spray versus typical oral LAO analgesics in a real-world setting, based on GPeR data for patients who had failed to achieve sufficient pain relief during recommended/established systemic therapy for severe NBP, thus prompting the start of a new analgesic treatment. Propensity score matching was used to control for several known demographic and clinical factors of pain, yielding a matched-pairs population, the majority of whom had chronic (61%) and severe dysfunctional (93%) pain. Patients had taken an average of about eight previous pain medications and, at baseline, were receiving an average of about four background pain medications. Comorbidities were frequent (approximately four comorbid conditions per patient).

An initial pre-planned analysis of the GPeR patient-reported data indicated noninferiority of NBX oromucosal spray vs oral LAO analgesics in relieving severe NBP during use in daily practice, as evidenced by a LS mean difference of −27.84% (*P* < 0.001) favoring NBX in the primary endpoint of change from baseline in ASR-9 scores. The subsequent pre-planned superiority analysis of the primary endpoint showed that NBX oromucosal spray was superior to LAO in relieving symptoms of NBP. In this regard, all secondary endpoints, i.e., the individual components of the ASR-9 measuring symptoms of pain and physical function and incorporating the well-established 0–100 VAS instrument used to measure pain intensity in RCTs, improved significantly from baseline in both treatment cohorts, with all between-group differences significantly favoring NBX oromucosal spray over LAO analgesics. In a subgroup analysis of patients with a high average 24-h pain intensity at baseline (VAS score ≥50 mm), it was found that significantly more patients treated with NBX than with LAO had improvement from baseline greater than or equal to the MCID or ≥50%, thus supporting the primary analysis.

The data align with and extend those from an earlier exploratory analysis of 12-week GPeR data involving 800 adults treated with add-on NBX oromucosal spray for severe chronic pain (nociceptive, mixed, or neuropathic pain) refractory to other analgesics [12]. That analysis was the first to report a change from baseline in ASR-9 scores as the primary endpoint. Approximately 30% of included patients had chronic LBP in this previous study. NBX oromucosal spray as add-on treatment was shown to be significantly better at providing overall symptom relief in patients with the neuropathic pain phenotype than in those with mixed or nociceptive pain (mean improvement in ASR-9 scores 54.9% vs 18.2% and −11.9%; both comparisons *P* < 0.001). A recent systematic review of pooled data from nine RCTs in patients with chronic neuropathic pain arising from various underlying conditions showed that add-on NBX oromucosal spray was more effective than background analgesia, with a small but discernible effect size (standardized mean difference of −0.21 in the 0–10 Numerical Rating Scale pain score) [21].

In the present study, patient-reported and physician-confirmed TRAE data reflected overall good tolerability for NBX oromucosal spray during 6 months’ observation, with a tolerability profile superior to that of LAO. Notably, significantly fewer patients treated with NBX spray than with LAO reported TRAEs or discontinued treatment because of TRAEs. With few exceptions, incidences of TRAEs were significantly lower in the NBX oromucosal spray cohort than in the LAO cohort.

Similar to all real-world registry analyses, this study is limited by its retrospective, nonrandomized observational design, although propensity score matching based on known relevant factors for pain allowed us to perform a comparison between two cohorts of patients with similar clinical features. Propensity score matching aims to even the distribution of baseline characteristics (covariates) between patient cohorts, in essence mimicking the effect of random treatment allocation in an RCT [31]. The predefined potential confounding factors matched in the present study are all established predictors of pain and known to be relevant for between-cohort comparisons. Nevertheless, a recognized drawback of propensity score matching is that the procedure can account only for observed (and observable) covariates and not for latent or “hidden” characteristics [32]. A further limitation is that the analysis did not account for the influence of any nonpharmacological treatments (e.g., physiotherapy) that may have been used during the observation time frame. Similar to the previous 12-week exploratory analysis of the GPeR database [12], the primary endpoint in the present analysis was the change from baseline in ASR-9 scores. Although the ASR-9 is not a scientifically developed and validated instrument, it is a composite measure of nine individual validated patient-reported tools that measure the impact of pain across multiple dimensions. In this regard, we believe that the ASR-9 may represent a more holistic approach to pain management, which is the cornerstone of routine clinical practice. Other strengths of the study are its large sample size and the inclusion of a population more representative of patients treated in pain units than those typically enrolled in RCTs. Moreover, because GPeR data are collected prospectively and systematically analyzed, the potential for missing values, as often occurs in retrospective studies, has been minimized. As participation in the GPeR is nationwide, our findings may have broad applicability.

Given the overall good tolerability and clinically relevant effectiveness of NBX oromucosal spray in providing relief from pain and related symptoms in patients with poorly responsive neuropathic LBP, as shown in this analysis, it seems appropriate to consider NBX as a useful alternative to commonly used oral LAO analgesics. This finding is particularly relevant in view of the link between prescription opioids and the growing opioid epidemic in several world regions [33], as well as limited evidence supporting the use of opioids in neuropathic pain [34]. Real-world evidence has a value of its own, reflecting patients’ evolution during treatment in usual daily practice, thus complementing the evidence derived from experimental conditions of selected participants in RCTs with smaller sample sizes. Nevertheless, our observations await confirmation in well-designed RCTs.

## Conclusion

Within the constraints of study limitations, such as the retrospective design and other potential sources of bias, this analysis of anonymized real-world data from a large propensity-matched GPeR sample showed that add-on treatment with NBX oromucosal spray was superior to and better tolerated than add-on treatment with typical oral LAO analgesics in patients with severe peripheral NBP inadequately controlled by recommended/established systemic therapy. Large well-designed prospective RCTs are warranted to confirm the effectiveness and tolerability of NBX oromucosal spray in patients with NBP. Meanwhile, prescribers can use the findings to decide for themselves whether a trial of add-on NBX in similar patients in clinical practice might be worthwhile.

## Authors’ Contributions

All authors contributed to data analysis, drafting, or revising the article; gave final approval of the version to be published; and agree to be accountable for all aspects of the work.

## Notes

Funding sources **:** Analyses were funded by an unrestricted grant from Almirall S.A. The concept for evaluating routine data collected via the GPeR was developed by Dr. Ueberall at the Institute of Neurological Sciences on behalf of the German Pain Association (Deutsche Gesellschaft für Schmerzmedizin) and German Pain League (Deutsche Schmerzliga). Its realization has been funded in part (∼25%) by an unrestricted scientific grant from Almirall Hermal, Germany. Neither Almirall, nor any of its employees, exerted any influence on data acquisition, the conduct of this analysis, or interpretation and publication of the results. The GPeR is hosted by an independent contract research organization by order of the German Pain Association, is under control of the Institute of Neurological Sciences, and has been collecting standardized real-world data from daily routine medical care since January 2000.

Disclosure and conflicts of interest: Drs. Ueberall and Mueller-Schwefe are physicians and independent of any significant/relevant financial or other relationship to the sponsor, apart from minor reimbursements for occasional lecture or consulting fees. Both are honorary members of the management boards of the German Pain Association and German Pain League. Dr. Ueberall has received financial support and/or expenses in form of research funds, consultancy fees, and/or renumerations for lecture activities from Allergan, Almirall, Amicus Therapeutics, Aristo Pharma, Bionorica, Glaxo Smith Kline, Grünenthal, Hapa Medical, Hexal, IMC, Kyowa-Kirin, Labatec, Mucos, Mundipharma, Nestle, Pfizer, Recordati, Servier, SGP-Pharma, Shionogi, Spectrum Therapeutics, Teva, and Tilray. Dr. Mueller-Schwefe has received financial support and/or expenses in the form of research funds, consultancy fees, and/or remunerations for lecture activities from Allergan, Almirall, Grünenthal, Mundipharma, Pfizer, PharmAllergan, ProStrakan, and Teva. Dr. Essner is a veterinarian and works as a scientific and medico-legal consultant for various pharmaceutical companies. Dr. Essner has received honoraria for consultancy services from Almirall Hermal GmbH and Granzer Regulatory Consulting & Service. Dr. Vila Silván is a physician and works as the Global Medical Advisor CNS for Almirall S.A. (Barcelona, Spain). Dr. Vila Silván is a full-time employee of Almirall S.A. commercialiser of Sativex across EU countries.

Study registration: European Network of Centres for Pharmacoepidemiology and Pharmacovigilance (ENCEPP), European Union Electronic Register of Post-Authorisation Studies (EU PAS Register) number EUPAS38969.
